# Neuropatía diabética y su relación con la calidad de vida en pacientes con diabetes tipo 2

**DOI:** 10.1016/j.aprim.2026.103454

**Published:** 2026-02-13

**Authors:** Jarek Ramón Arévalo Ramírez, Daniela Leticia Castañón Sánchez, Oscar Isaías Vargas Ordoñez, Hilda Sara Camarena Velázquez, María del Carmen Aguirre García, Mónica Catalina Osorio Granjeno

**Affiliations:** a Instituto Mexicano del Seguro Social, Unidad de Medicina Familiar No. 33, Azcapotzalco, Ciudad de México, México; b Instituto Mexicano del Seguro Social, Unidad de Medicina Familiar No. 33, Educación e Investigación en Salud, Azcapotzalco, Ciudad de México, México; c Instituto Mexicano del Seguro Social, Unidad de Medicina Familiar No. 33, Consulta externa de Medicina Familiar, Azcapotzalco, Ciudad de México, México

**Keywords:** Diabetes mellitus tipo 2, Neuropatía diabética, Calidad de vida, Factores de riesgo, Type 2 diabetes mellitus, Diabetic neuropathy, Quality of Life, Risk Factors

## Abstract

**Objetivo:**

Determinar la relación entre la neuropatía diabética (ND) y la calidad de vida (CV) en pacientes con diabetes mellitus tipo 2 (DM2).

**Emplazamiento:**

Unidad de Medicina Familiar No. 33 (UMF 33), El Rosario, Azcapotzalco, Ciudad de México, México.

**Diseño:**

Estudio observacional, analítico, transversal.

**Participantes:**

365 personas mayores de 18 años con diagnóstico de DM2, adscritas a un programa especializado en atención a la diabetes.

**Mediciones:**

Se aplicó un cuestionario sociodemográfico y dos instrumentos estandarizados: el *Michigan Neuropathy Screening Instrument* (MNSI), en sus componentes cuestionario (MNSIQ) y exploración física (MNSIEF) y el cuestionario SF-12v2 para evaluar la CV en sus componentes físico (PCS) y mental (MCS). Se analizaron frecuencias y porcentajes. Para la estadística inferencial se empleó la prueba de chi cuadrada.

**Resultados:**

De acuerdo con el MNSIQ, 35,6% presentó signos de ND, mientras que en el MNSIEF detectó signos de ND en el 99,2% de los participantes. El 59,5% reportó buena CV física, mientras que el 61,4% indicó mala CV mental. A pesar de las diferencias descriptivas observadas, el análisis estadístico no logró demostrar asociaciones significativas entre ND y CV.

**Conclusiones:**

Se encontró una discordancia entre la percepción de los pacientes y la exploración física. Los hallazgos sugieren que la ND podría tener un impacto negativo en la CV, especialmente en el componente mental (MCS). La detección activa de ND en el primer nivel de atención podría contribuir a una atención más integral y mejorar la percepción de bienestar en personas con DM2.

## Abstract (en)

**Objective:**

To determine the association between diabetic neuropathy (DN) and quality of life (QoL) in patients with type 2 diabetes mellitus (DM2).

**Setting:**

Family Medicine Unit No. 33 (UMF 33), El Rosario, Azcapotzalco, Mexico City, Mexico.

**Design:**

Observational, analytical, cross-sectional.

**Participants:**

365 participants aged 18 years or older with a diagnosis of DM2, ascribed to a specialized diabetes care program.

**Measurements:**

A sociodemographic questionnaire and two standardized instruments were applied: the Michigan Neuropathy Screening Instrument (MNSI), in its questionnaire (MNSIQ) and physical examination (MNSIEF) components and the SF-12v2 questionnaire to assess QoL through its physical (PCS) and mental (MCS) components. Frequencies and percentages were analyzed. The chi-square test was used for inferential statistics.

**Results:**

According to the MNSIQ, 35.6% presented signs of ND, while the MNSIEF it was detected in 99.2% of the participants. 59.5% reported good physical QoL, while 61.4% reported poor mental QoL. Although descriptive differences were notable, the statistical power was limited to demonstrate consistent associations between ND and QoL.

**Conclusions:**

The findings suggest a discordance between patient self-perception and physical examination. ND could have a negative impact on QoL, especially on the mental component (MCS). Active screening for ND in primary care settings may enhance comprehensive care and improve perceived well-being in people with DM2.

## Introducción

La diabetes mellitus tipo 2 (DM2) representa un problema de salud pública a nivel mundial. En México, afecta a más de 12 millones de personas^1^, constituyéndose como una de las enfermedades de mayor prevalencia tanto en el ámbito institucional como en el sector privado. Su impacto abarca diversas esferas, principalmente la metabólica, afectando considerablemente la calidad de vida de quienes la padecen, debido a sus complicaciones crónicas, entre las cuales destaca la neuropatía diabética periférica (NDP)^2^, ^3^. Se estima que hasta el 50% de los pacientes con DM2 desarrollarán algún grado de NDP^4^, ^5^, ^6^. Esta complicación se caracteriza por síntomas como dolor, disestesias y pérdida de la sensibilidad, lo cual incrementa el riesgo de desarrollar úlceras y, en casos avanzados, puede culminar en amputaciones, particularmente de extremidades inferiores. Por ello, su evaluación periódica resulta fundamental para la detección oportuna y el manejo integral de los pacientes diabéticos.

Diversos estudios^5^, ^7^, ^8^ han documentado que la NDP no solo limita la funcionalidad física, sino que también deteriora de manera significativa la esfera emocional. Esta afectación multidimensional influye directamente en la calidad de vida (CV), la cual debe entenderse desde una perspectiva tanto física como mental. A pesar de la relevancia clínica de esta complicación, la evidencia nacional relacionada con su impacto en la calidad de vida sigue siendo limitada^9^, ^10^ lo que refuerza la necesidad de generar conocimiento desde el primer nivel de atención.

Actualmente, uno de los instrumentos más utilizados y validados para el cribaje de NDP es el MNSI, el cual integra una sección de autoevaluación y otra de exploración física^11^, ^12^, ^13^. Por otro lado, la CV puede evaluarse mediante el cuestionario SF-12v2, que permite valorar de forma resumida los componentes Physical Component Summary (PCS) y Mental Component Summary (MCS), mostrando utilidad en población mexicana^14^, ^15^.

El objetivo de esta investigación fue determinar la relación entre la NDP y la CV en pacientes con DM2 adscritos a un programa especializado en el seguimiento de personas con DM2, en una unidad de medicina familiar de una institución pública del sector salud.

## Material y métodos

**Figura 1:**
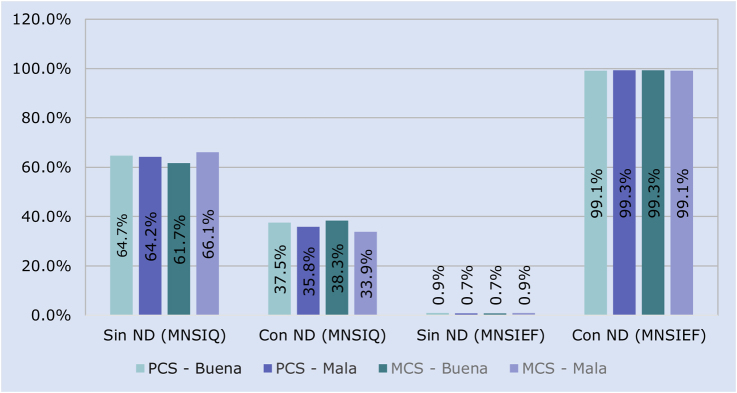
Neuropatía diabética (cuestionario y exploración física de Michigan) vs. calidad de vida (componentes físico y mental). Fuente: elaboración propia. ND: neuropatía diabética; MNSIQ: Cuestionario de Michigan; MNSIEF: Exploración física de Michigan.; PCS: componente físico; MCS: componente mental.

Se realizó un estudio observacional, analítico, transversal durante el mes de febrero de 2024, en la Unidad de Medicina Familiar No. 33, ubicada en El Rosario, Azcapotzalco, Ciudad de México (fig. 1). La población de estudio estuvo conformada por 365 pacientes mayores de 18 años con diagnóstico de DM2, adscritos a un modelo institucional especializado en su seguimiento durante el mes de febrero 2024 que aceptaron participar mediante consentimiento informado. Se excluyeron pacientes con diagnóstico reciente (< 3 meses), alteraciones neuromusculares (síndrome de Guillain-Barré, secuelas de poliomielitis), complicaciones avanzadas (pie de Charcot, pie diabético en tratamiento, amputaciones), insuficiencia venosa grado CEAP 4-6, deformidades articulares por artritis reumatoide y aquellos en terapia ostomal.”. El muestreo fue no probabilístico por conveniencia.

La recolección de datos se llevó a cabo mediante una cédula sociodemográfica que incluyó variables como edad, escolaridad, ocupación, estado civil, peso, talla, índice de masa corporal (IMC) y la glucemia en ayuno, medida por Dextrostix (DXTX) (Anexo 1). Se utilizaron dos instrumentos: el MNSI, validado para población mexicana, que se aplicó en sus dos componentes: el MNSIQ y MNSIEF, que incluyó evaluación del reflejo aquíleo, sensibilidad con monofilamento de 10 g, percepción vibratoria con diapasón de 128 Hz, inspección de pies y fuerza motora distal. Se consideró presencia de neuropatía si el puntaje era ≥ 7 en el cuestionario o ≥ 2 en la exploración física. El instrumento mostró un alfa de Cronbach de 0,84 (Anexo 2)^7^. Para la evaluación de la CV se empleó el cuestionario SF-12v2, que valora dos dimensiones: el PCS y MCS (Anexo 3). Los resultados se interpretaron conforme a los valores normativos; una puntuación inferior a 50 representa mala calidad de vida, mientras que valores iguales o superiores indican buena calidad de vida. El cuestionario ha sido validado en población mexicana, con alfa de Cronbach de 0,63 para PCS y 0,72 para MCS^8^. Ambos instrumentos se seleccionaron por su validez en población mexicana y su utilidad clínica para evaluar neuropatía periférica y calidad de vida, respectivamente.

El análisis estadístico se realizó con el programa IBM SPSS Statistics versión 28 (IBM Corp., Armonk, NY, Estados Unidos). Se aplicó estadística descriptiva mediante frecuencias y porcentajes para variables cualitativas y media con desviación estándar o mediana con rango intercuartílico para variables cuantitativas, según su distribución. Para evaluar la asociación entre ND y CV se utilizó la prueba de chi cuadrada, considerando significativo un valor de p < 0,05.

El estudio fue aprobado por el Comité Local de Investigación en Salud. Todos los participantes firmaron un consentimiento informado, en apego a los principios éticos de la investigación biomédica y las normativas nacionales vigentes.

**Figure:**
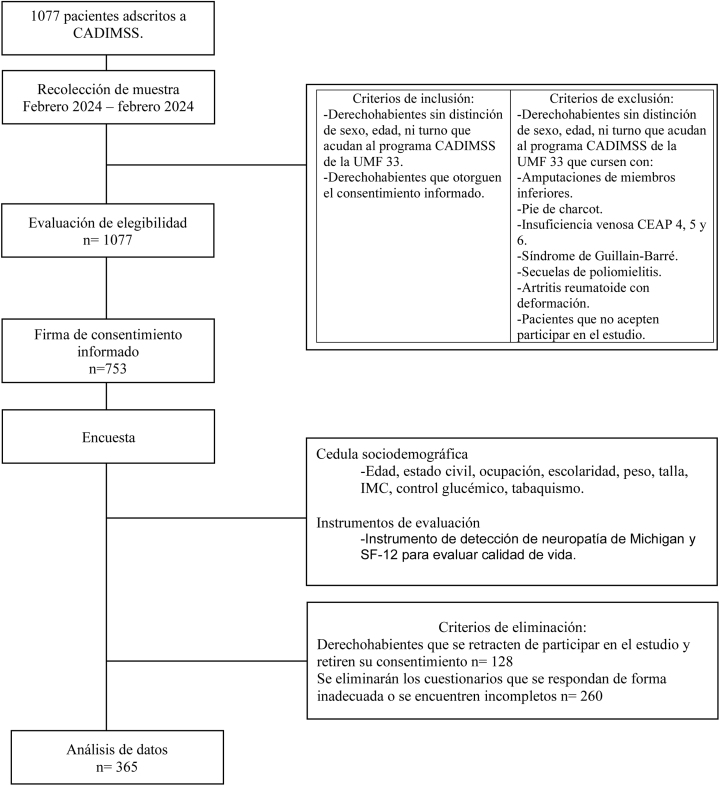
**Esquema general del estudio**.

## Resultados

La muestra incluyó 365 participantes. La edad media fue de 60,71 años (DE ± 10). En cuanto a las variables antropométricas, la mediana de peso fue de 70 kg (RIC: 62,24–81,06), y la talla presentó una mediana de 1,56 m (RIC: 1,51–1,64), la glucemia en ayuno, medida por DXTX, tuvo una mediana de 130 mg/dL (RIC: 115–152), y el IMC fue de 28,83 kg/m^2^ (RIC: 25,97–33,29).

Respecto al turno de atención, el 52,6% fue en el vespertino. La ocupación más frecuente fue el trabajo en el hogar con 38,4%.

En relación con el nivel educativo, el 32,9% tenían educación secundaria. El 12,9% de los participantes eran fumadores y el 45,8% estaban casados (tabla 1).

**Tabla 1:** Características basales de la población de CADIMSS de la UMF 33

Variables	N = 365
*Edad (años) Media, DE*	60,71 ± 10
*Peso (kg) Mediana, RIC*	70 (62,24-81,06)
*Talla (m) Mediana, RIC*	1,56 (1,51-1,64)
*Glucemia en ayuno (mg/dl) (DXTX) Mediana, RIC*	130 (115-152)
*IMC (kg/m*^*2*^*) Mediana RIC*	28,83 (25,97-33,29)
*Turno No. (%)*	
Matutino	173 (47,4)
Vespertino	192 (52,6)
*Ocupación No. (%)*	
Hogar	140 (38,4)
Informal	94 (25,8)
Jubilado/Pensionado	72 (20.0)
*Escolaridad No. (%)*	
Primaria	91 (24,9)
Secundaria	120 (32,9)
Preparatoria	94 (25,8)
*Tabaquismo No. (%)*	
No	318 (87,1)
Sí	47 (12,9)
*Estado civil No. (%)*	
Casado	167 (45,8)
Soltero	61 (16,7)
Viudo	55 (15,1)

N: número total de la muestra; No.: número; %: porcentaje; DE: desviación estándar; RIC: rango intercuartílico; DXTX: Dextrostix.

Fuente: cédula de recolección de datos «Asociación entre calidad de vida, factores de riesgo y neuropatía diabética en CADIMSS de la UMF 33».

El 64,4% de los participantes reportaron no tener ND según el MNSIQ, mientras que el 35,6% sí presentaban esta condición. En cuanto al MNSIEF, el 99,2% de los participantes presentaban ND, y solo el 0,8% no la tenían (tabla 2).

**Tabla 2:** Neuropatía diabética en CADIMSS de la UMF 33

N = 365		
	Sin neuropatía	Con neuropatía
	No. (%)	No. (%)
MNSIQ	235 (64,4)	130 (35,6)
MNSIEF	3 (0,8)	362 (99,2)

N: número total de la muestra; No.: número; MNSIQ: Cuestionario de Michigan; %: porcentaje; MNSIEF: Exploración física de Michigan.

Fuente: cédula de recolección de datos «Asociación entre calidad de vida, factores de riesgo y neuropatía diabética en CADIMSS de la UMF 33».

En el PCS, el 59,5% de los participantes obtuvo una puntuación correspondiente a buena CV, mientras que el 40,5% la calificaron como mala. En cuanto al MCS, el 38,6% de los participantes reportó buena CV y el 61,4% presentó una mala CV (tabla 3). En relación con el MNSIQ, entre los participantes sin ND, el 64,7% se percibía con buena CV en el PCS y el 61,7% en el MCS. En contraste, entre quienes sí se percibían con ND, solo el 37,5% refirió buena CV en el PCS y el 38,3% en el MCS.

**Tabla 3:** Calidad de vida en CADIMSS de la UMF 33

N = 365		
	Buena calidad de vida	Mala calidad de vida
	No. (%)	No. (%)
PCS	217 (59,5)	148 (40,5)
MCS	141 (38,6)	224 (61,4)

N: número total de la muestra; No.: número; %: porcentaje; PCS: componente físico; MCS: componente mental.

Fuente: cédula de recolección de datos «Asociación entre calidad de vida, factores de riesgo y neuropatía diabética en CADIMSS de la UMF 33».

A pesar de estas diferencias, no se encontró una asociación estadísticamente significativa entre la presencia de ND y la CV, tanto en el PCS (X^2^ = 0,004, p = 0,999) como en el MCS (X^2^ = 0,720, p = 0,999). Cabe destacar que, incluso entre los participantes sin percepción de ND según el MNSIQ, una proporción considerable (66,1%) reportó mala CV en el componente mental (MCS), lo que sugiere la influencia de otros factores psicosociales no evaluados en este estudio. En cuanto a los hallazgos obtenidos mediante la MNSIEF, no se identificó una asociación estadísticamente significativa entre la ND y la CV, en el PCS (X^2^ = 0,065, p = 0,999) ni en el MCS (X^2^ = 0,036, p = 0,999); sin embargo, en la distribución de frecuencias se observó que entre los participantes sin ND, el 0,7% reportó mala CV en el PCS y el 0,9% en el MCS; mientras que en el grupo con ND confirmada por el MNSIEF, estas proporciones aumentaron notablemente a 99,3% y 99,1%, respectivamente (fig. 1).

## Discusión

Este estudio evaluó la asociación entre ND y CV en adultos que acudían a un programa especializado en atención de diabetes de una clínica de primer nivel de atención del IMSS en El Rosario, Azcapotzalco, Ciudad de México. Se identificó una alta prevalencia de ND en la población estudiada (99,2%), especialmente al aplicar métodos de exploración física sistematizada, lo cual sugiere un subdiagnóstico significativo, cuando se utilizan únicamente herramientas subjetivas. Gad et al.^9^ (2024) reportan que hasta el 80% de los casos de neuropatía periférica permanecen sin diagnóstico, especialmente en contextos de atención primaria sobrecargada. Este hallazgo es consistente con los resultados del presente estudio.

Este subdiagnóstico ha sido ampliamente documentado en países como Arabia Saudita, Qatar, Kuwait, Alemania, Reino Unido, EE. UU., Japón, Malasia, Hong Kong, Filipinas, Taiwán y Tailandia; la NP puede estar sin diagnóstico en un rango que va del 35% al 99,8%.^9^ Lo anteriormente planteado puede deberse a la normalización de síntomas, uso crónico de analgésicos y el tiempo limitado en consulta, lo que dificulta una exploración dirigida. De igual forma, esta situación ha sido reportada por Atmaca et al. (2024), quienes identificaron una discrepancia notable entre la sintomatología referida y los hallazgos clínicos: el 45,9% de los pacientes con exploración física positiva no reportaban síntomas, evidenciando una alta prevalencia de neuropatía subclínica; en contraste, solo el 17,6% de quienes presentaban síntomas tenían hallazgos positivos, lo que resalta que la exploración física es más sensible para detectar casos tempranos de ND^10^.

La ND se evaluó con el MNSI, resultando útil como herramienta práctica de tamizaje en el primer nivel de atención. Beshyah et al. (2024) reportan que el MNSI alcanza una sensibilidad del 80% y especificidad del 95% para el diagnóstico de NDP, facilitando la identificación tanto de casos clínicamente manifiestos como de formas subclínicas^16^, mientras que otros estudios, como Mogilevskaya et al. (2024) y Matheson et al. (2021) han señalado que los instrumentos subjetivos como el MNSIQ pueden subestimar la prevalencia real de ND, especialmente en etapas iniciales con síntomas vagos o ausentes. En este contexto, la exploración física cobra especial relevancia: al incluir maniobras como el uso del monofilamento de 10 g, el diapasón de 128 Hz y la evaluación del reflejo aquileo, ha demostrado que incrementa la tasa de detección, incluso en pacientes sin sintomatología referida 17, 18. De manera similar Carmichael et al. (2021), respaldan este enfoque al proponer la implementación de estrategias combinadas de tamizaje subjetivo y físico como modelo costo-efectivo para el primer nivel de atención, capaz de detectar casos subclínicos y mejorar los desenlaces clínicos en poblaciones de alto riesgo^19^.

En el presente estudio, ambos componentes del MNSI fueron aplicados por el mismo investigador, siguiendo un protocolo estandarizado, por lo que consideramos poco probable un sesgo sistemático de aplicación. La discrepancia observada pudiera deberse a que muchos pacientes no reconocen sus síntomas neuropáticos, a diferencia de los signos objetivos detectados en la exploración física, lo que refuerza la necesidad de no basar el cribado únicamente en el autoinforme de síntomas.

Se evidenció una alta proporción de mala CV en el MCS (61,4%), lo que resalta la necesidad de integrar de forma sistemática el abordaje emocional en el manejo del paciente con ND. Este hallazgo refuerza la pertinencia del enfoque biopsicosocial del médico familiar, especialmente en contextos de dolor crónico. Kovačević et al. (2024) señalan que factores psicosociales como la depresión, la ansiedad, las creencias disfuncionales y el aislamiento social influyen significativamente en la experiencia del dolor y en los resultados clínicos, por lo que proponen un modelo de atención multidisciplinario e integrado que contemple el componente emocional como parte esencial del tratamiento^20^. En la misma línea, Degu et al. (2019) reportaron que los pacientes con dolor neuropático presentan una disminución significativa en todos los dominios de un instrumento estandarizado que evalúa la calidad de vida, siendo el componente mental el más afectado^21^. Por su parte, Moghadam et al. (2025) identificaron que variables como el dolor mental, la autocompasión y la expresividad emocional predicen de forma significativa tanto la CV como las conductas de autocuidado, evidenciando que el sufrimiento emocional tiene un impacto clínico tangible que debe ser abordado desde un enfoque integral^22^.

No se encontró una asociación significativa entre los factores de riesgo clínicos comúnmente evaluados, como la edad, el tiempo de evolución de la DM2 o el control glucémico y la CV, lo que sugiere que otros elementos del modelo biopsicosocial como la salud mental, el apoyo familiar, el nivel socioeconómico y las comorbilidades podrían influir más en la percepción subjetiva del bienestar. Borbjerg et al. (2025) demostraron que tanto la neuropatía diabética dolorosa como la indolora se asocian con una reducción significativa en los puntajes de un instrumento estandarizado que evalúa la CV relacionada con la salud, incluyendo aspectos como el bienestar emocional, la función física y la interacción social. Asimismo, los pacientes con neuropatía presentaron mayores niveles de ansiedad y depresión que aquellos sin esta complicación. Estas asociaciones se mantuvieron incluso después de ajustar por variables clínicas (como control glucémico, duración de la diabetes o presencia de otras complicaciones) y demográficas (edad, sexo y nivel educativo), lo que indica que la neuropatía afecta negativamente la CV de forma independiente^23^. Si bien en el presente estudio no se realizaron ajustes multivariados, se observó una tendencia similar: los participantes con ND presentaron una percepción más deteriorada de su CV, especialmente en los PCS (99,3%) y MCS (99,1%) del SF-12v2.

De acuerdo con los Estándares de Atención 2025 de la *American Diabetes Association* enfatizan que la evaluación médica debe ir más allá de los parámetros metabólicos, integrando el cribado sistemático de trastornos afectivos, limitaciones funcionales y barreras sociales, con el fin de individualizar el tratamiento y mejorar los desenlaces en salud^23^.

Estos hallazgos tienen implicaciones relevantes para la práctica clínica en el primer nivel de atención, ya que refuerzan la necesidad de establecer protocolos estructurados de cribado para la ND, así como intervenciones dirigidas a mejorar el bienestar emocional, especialmente en contextos urbanos de alta carga, como los servicios del IMSS.

Dado que la CV en pacientes con ND está influida por factores biopsicosociales como la salud mental, el entorno familiar y la situación socioeconómica, el abordaje debe ser integral, centrado en la persona y adaptado al contexto local. En este sentido, los resultados del estudio pueden orientar el desarrollo de estrategias comunitarias y de protocolos de atención diferenciada, ajustados al nivel de afectación funcional y emocional, lo que contribuiría a optimizar recursos y mejorar los resultados clínicos y psicosociales.

Este estudio también subraya la necesidad de promover la educación para el autocuidado desde el diagnóstico de la DM2, con énfasis en el empoderamiento del paciente y en el rol del médico familiar como educador, fomentando estilos de vida saludables, adherencia al tratamiento y detección oportuna de complicaciones.

Este estudio presenta algunas limitaciones que deben considerarse al interpretar los resultados. En primer lugar, el diseño transversal impide establecer relaciones causales entre la ND, los factores biopsicosociales y la CV, limitando las inferencias a asociaciones observacionales. Asimismo, el uso de instrumentos generales como el SF-12v2, si bien validados, pueden no captar con suficiente sensibilidad las particularidades psicoemocionales específicas de esta población, especialmente en lo relacionado con el dolor neuropático y sus implicaciones funcionales.

En cuanto a las variables, no se incluyeron comorbilidades psiquiátricas diagnosticadas formalmente, ni se aplicaron escalas específicas de salud mental, lo cual puede subestimar la carga emocional real del paciente con ND. Del mismo modo, aspectos como la red de apoyo o el nivel de resiliencia personal que influyen en la percepción de bienestar y afrontamiento de la enfermedad no fueron evaluados de manera estructurada, representando un área de oportunidad para estudios posteriores.

Una limitación relevante es la ausencia de modelos multivariados: la muy alta prevalencia de ND según el MNSIEF (99,2%), junto con el número reducido de participantes sin ND, impidió obtener estimaciones estables en regresión logística, por lo que solo se presentan asociaciones bivariadas.

Finalmente, el ingreso al Centro de Atención a la Diabetes en el Instituto Mexicano del Seguro Social (CADIMSS) pudo haber generado sesgo de selección al incluir principalmente a pacientes con mayor afectación clínica, y la limitación de tiempo en consulta pudo haber afectado la calidad de algunos datos recabados.

## Conclusión

Sería recomendable que el médico de familia realice una exploración física sistemática utilizando herramientas accesibles como el MNSI antes de referenciar a CADIMSS, así como evaluar el componente emocional mediante instumentos rápidos como es el SF-12v2, especialmente en pacientes con mayor vulnerabilidad social. Asimismo, se sugiere capacitar al personal de salud en detección temprana y fomentar la educación para el autocuidado desde el diagnóstico, posicionando al médico familiar como figura clave en la prevención de complicaciones. Finalmente, se propone implementar intervenciones complementarias como talleres de afrontamiento emocional y grupos de apoyo que favorezcan la resiliencia y reduzcan el impacto psicosocial de la ND.

## Financiación

La presente investigación no ha recibido ayudas específicas provenientes de agencias del sector público, sector comercial o entidades con fines de lucro. El estudio no contó con apoyo financiero.

## Consideraciones éticas

El estudio fue aprobado por el Comité Local de Ética en Investigación del Instituto Mexicano del Seguro Social con el número de registro R-2023-3404-125 y se obtuvo consentimiento informado de los participantes, de acuerdo con la Declaración de Helsinki.

## Conflictos de interès

Los autores no tienen conflicto de intereses que declarar.

**Lo conocido sobre el tema**

- La neuropatía diabética es una complicación frecuente y subdiagnosticada en personas con diabetes mellitus tipo 2.
- La calidad de vida en estos pacientes puede verse comprometida tanto en su dimensión física como mental.
- La mayoría de los estudios se han centrado en mediciones clínicas, con poca exploración del componente percibido por el paciente.

**Qué aporta este estudio**

- Identifica una discrepancia relevante entre la percepción subjetiva de neuropatía y los hallazgos en la exploración física.
- Evidencia un mayor impacto en la calidad de vida mental que en la física entre los pacientes evaluados.
- Refuerza la necesidad de implementar estrategias activas de cribaje para neuropatía en el primer nivel de atención.
