# Supplementary material for: Diabetic neuropathy and its relationship with quality of life in patients with type 2 diabetes
Source: Aten Primaria. 2026 Feb 13;58(5):103454. [Article in Spanish] doi: 10.1016/j.aprim.2026.103454 (PMC12925270; doi:10.1016/j.aprim.2026.103454)
Supplement: Supplementary file 1 [file mmc1.pdf]

Material suplementario

Anexo 1: CUESTIONARIO SOCIODEMOGRÁFICO

Instrucciones: Responda las siguientes preguntas y marque con una X de acuerdo con su situación donde corresponda, datos como peso, talla, última cifra de glucosa en sangre déjelos en blanco.

1. ¿Qué edad tiene? _____________ años

2. ¿Cuál es su ocupación?

Hogar ( ) Estudiante ( ) Informal ( ) Profesionista ( ) Jubilado/pensionado ( ) Desempleado ( )

3. ¿Qué escolaridad tiene?

No estudié ( ) Primaria ( ) Secundaria ( ) Preparatoria ( ) Licenciatura ( ) Posgrado ( )

4. Fuma

Sí ( ) No ( )

5. Estado civil

Soltero ( ) Casado ( ) Unión libre ( ) Viudo ( ) Separado/divorciado ( )

Peso: kg
Talla: mts.
Utlima cifra de glucosa en sangre: mg/dl IMC Kg/m2

Fuente: elaboración propia.

Anexo 2: INSTRUMENTO DE TAMIZAJE DE NEUROPATÍA MICHIGAN

**A. Historia. (Debe completarse por las personas con Diabetes). Por favor tome unos minutos para contestar las siguientes preguntas sobre la sensación en las piernas y los pies. Marque sí o no en función de cómo se sienten generalmente. Gracias.**	SI	NO
¿Ha notado sus piernas o pies entumecidos?		
¿Alguna vez ha tenido dolor o ardor en los pies y/o piernas?		
¿Son sus pies demasiado sensibles al tacto?		
¿Le dan calambres musculares en las piernas o en los pies?		
¿Alguna vez ha tenido sensación de picor en las piernas o en los pies?		
¿Le duele cuando la ropa de cama toca su piel?		

¿Cuándo se baña usted es capaz de detectar el agua caliente de la fría?		
¿Ha tenido alguna vez una herida abierta en el pie?		
¿Su médico le ha dicho que usted tiene neuropatía diabética?		
¿Se siente débil durante la mayor parte del tiempo?		
¿Sus síntomas empeoran por la noche?		
¿Le duelen las piernas cuando camina?		
¿Es usted capaz de sentir los pies cuando camina?		
¿La piel de sus pies es tan seca que se agrieta?		
¿Alguna vez ha tenido una amputación?		
TOTAL		

B. EXPLORACIÓN FÍSICA

**Apariencia de los pies.**	**Derecho**			**Izquierdo**		
	Normal	Si	No	Normal	Si	**No**
**Marque lo que corresponda**	Deformidades			Deformidades		
	Piel Seca			Piel Seca		
	Callos			Callos		
	Infección			Infección		
	Fisuras			Fisuras		
	Otros:			Otros:		
**Ulceraciones**	Ausente	Presente		Ulceraciones	Ausente	Presente
**Reflejos**	Presente	Presente/ Reforzamiento	Ausente	Presente	Presente/ Reforzamiento	Ausente
**Vibración o percepción del dedo gordo del pie.**	Presente	Disminuido	Ausente	Presente	Disminuido	Ausente
**Monofilamento**	Presente	Disminuido	Ausente	Presente	Disminuido	Ausente

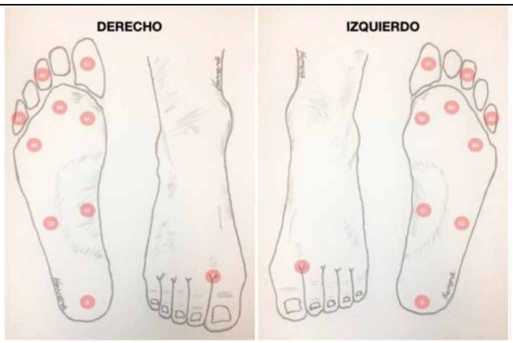
**Total:**

Fuente: elaboración propia.

Anexo 3: CUESTIONARIO "SF-12" SOBRE EL ESTADO DE SALUD

**INSTRUCCIONES: Las preguntas que siguen se refieren a lo que usted piensa sobre su salud. Sus respuestas permitirán saber cómo se encuentra usted y hasta qué punto es capaz de hacer sus actividades habituales.**<br>**Por favor, conteste cada pregunta marcando una casilla. Si no está seguro/a de cómo responder a una pregunta, por favor, conteste lo que le parezca más cierto.**

1. En general, usted diría que su salud es:

Excelente ( ) Muy buena ( ) Buena ( ) Regular ( ) Mala ( )

Las siguientes preguntas se refieren actividades o cosas que usted podría hacer en un día normal. Su salud actual ¿le limita para hacer esas actividades o cosas? Si es así ¿Cuánto?

2. Esfuerzos moderados: como mover una mesa pasar la aspiradora, jugar a los bolos o caminar más de 1 hora.
Si. Me limita mucho. ( )
Si me limita un poco. ( )
No, no me limita nada. ( )

3. Subir varios pisos por la escalera.
Sí. Me limita mucho. ( )
Si me limita un poco. ( )
No, no me limita nada. ( )

Durante las últimas 4 semanas ¿ha tenido alguno de los siguientes problemas en su trabajo o en sus actividades cotidianas, a causa de su salud física

4. ¿Hizo menos de lo que hubiera querido hacer?
SI. ( )
NO. ( )

5. ¿Tuvo que dejar de hacer algunas tareas en su trabajo o en sus actividades
SI. ( )

NO. ( )

Durante las últimas 4 semanas ¿ha tenido alguno de los siguientes problemas en su trabajo o en sus actividades cotidianas a causa de algún problema emocional (como estar triste, deprimido o nervioso)?

1. ¿Hizo menos de lo que hubiera querido hacer por algún problema emocional?
SI. ( )
NO. ( )

2. ¿No hizo su trabajo o sus actividades cotidianas tan cuidadosamente como de costumbre por algún problema emocional?
SI. ( )
NO. ( )

3. Durante las últimas 4 semanas ¿hasta qué punto el dolor ha dificultado su trabajo habitual (incluido el trabajo fuera de casa y las tareas domésticas)?

NADA. ( ) UN POCO. ( ) REGULAR. ( ) BASTANTE. ( ) MUCHO. ( )

Las preguntas que siguen se refieren a como se ha sentido y como le han ido las cosas durante las 4 últimas semanas. En cada pregunta responda lo que se parezca más a como se ha sentido usted. Durante las 4 últimas semanas ¿cuánto tiempo....

4. .. se sintió calmado y tranquilo?

Siempre. ( ) Casi siempre. ( ) Muchas veces. ( ) Algunas veces. ( ) Solo alguna vez. ( ) Nunca ( )

5. .. tuvo mucha energía?

Siempre. ( ) Casi siempre. ( ) Muchas veces. ( ) Algunas veces. ( ) Solo alguna vez. ( ) Nunca ( )

6. ..se sintió desanimado y triste?

Siempre. ( ) Casi siempre. ( ) Muchas veces. ( ) Algunas veces. ( ) Solo alguna vez. ( ) Nunca ( )

7. Durante las últimas 4 semanas ¿con que frecuencia la salud física o los problemas emocionales le han dificultado sus actividades sociales (como visitar a los amigos o familiares)?

Siempre. ( ) Casi siempre. ( ) Muchas veces. ( ) Algunas veces. ( ) Solo alguna vez. ( ) Nunca ( )

¡GRACIAS POR SU PARTICIPACIÓN!

Fuente: elaboración propia.
